# A Comparative View on Easy to Deploy non-Integrating Methods for Patient-Specific iPSC Production

**DOI:** 10.1007/s12015-015-9619-3

**Published:** 2015-09-05

**Authors:** Stefano Manzini, Leena E. Viiri, Suvi Marttila, Katriina Aalto-Setälä

**Affiliations:** BioMediTech, University of Tampere, 33014 Tampere, Finland; Department of Pharmacological and Biomolecular Sciences, Università degli Studi di Milano, Milan, Italy; Heart Hospital, Tampere University Hospital, Tampere, Finland

**Keywords:** Induced pluripotent stem cells (iPSC), Reprogramming, Human dermal fibroblast, Episomal, Plasmid, Electroporation

## Abstract

Induced pluripotent stem cells (iPSCs) are routinely produced from dermal fibroblasts, with potential applications ranging from in vitro disease models to drug discovery and regenerative medicine. The need of eliminating the remaining reprogramming factors after iPSC production spurred the development of non-integrating viruses such as Sendai and other methods to deliver episomal vectors, which are progressively lost upon cell division. We compared four widespread methods (Sendai virus, Nucleofector, Neon transfection system and Lipofectamine 3000) to generate integration-free iPSC lines from primary human dermal fibroblasts (hDF) of three patients. Furthermore, we performed extensive characterization of the iPSC lines. We were able to produce iPSC lines with all tested methods with variable efficiency. Sendai virus method achieved the overall highest reprogramming rate, followed by electroporation-based methods Nucleofector and Neon transfection systems. Chemical-based Lipofectamine 3000 delivery resulted in the lowest number of iPSC colonies. We found the reprogramming rate to be intrinsically dependent on the individual hDFs but the amenability of each hDF to reprogramming showed consistency between methods. Regardless of the reprogramming strategy, iPSCs obtained did not reveal any significant differences in their morphology, expression of pluripotency markers, EB formation, karyotype or gene expression profiles.

**Electronic supplementary material:**

The online version of this article (doi:10.1007/s12015-015-9619-3) contains supplementary material, which is available to authorized users.

## **Introduction**

Induced pluripotent stem cells (iPSCs), which show an extensive capability for self-renewal, reproduction and differentiation potential overlapping that of embryonic stem cells (ESCs) can be generated from terminally differentiated cells with a defined cocktail of chemical and/or genetic factors [1]. Patient-specific iPSCs hold enormous potential for disease modeling, cell-based therapies and possibly future organ-based therapies [2–4].

Early methods of iPSC production resulted in the random integration into the host genome of the exogenous reprogramming vectors in iPSCs, leading to increased tumor formation and mortality in mice generated from these cells, raising concerns about safety [5]. To tackle them, safer methods have been developed which make use of episomal plasmids that can be delivered by non-integrating viruses (Sendai), electroporation or chemical methods [6]. Residual safety concerns and cost-related issues of virus-based reprogramming still make the use of simpler methods attractive. These methods rely on electrical current or chemical compounds to deliver the reprogramming factors into somatic cells. They are reportedly less efficient in reprogramming but can be employed more broadly and do not require dedicated laboratory space and Biosafety Committee review and approval. In the present report we focused on four popular methods (Sendai virus, Nucleofector and Neon Electroporation Systems, and Lipofectamine 3000), which are widely employed in laboratories, and compared their efficiency in reprogramming patient-derived primary human dermal fibroblasts (hDF) into iPSCs. All methods were used to reprogram hDFs originating from three different patients to take variability into account. Finally, we also characterized the iPSC lines for pluripotency by PCR, immunocytochemistry and embryoid body (EB) formation, as well as for karyotype.

## Materials and Methods

### Isolation of Dermal Fibroblasts and Cell Culture

A skin biopsy of approximately 3 mm was collected from the patients (Ethical approval number R12123) by a trained health care professional. Primary human dermal fibroblasts (hDFs) were obtained by explantation from the skin samples and the cells were grown in Fibroblast Medium, containing DMEM (Life Technologies) supplemented with 10 % FBS (Sigma Aldrich), penicillin/streptomycin and L-glutamine (Lonza), medium was refreshed every 2–3 days and fibroblasts were split 1:2–1:4 when they reached ca. 80 % confluence.

Established colonies were cultured in knockout serum replacement (KSR) medium using mouse embryonic fibroblasts (MEF; Millipore, Billerica, MA) as feeders. The components of KSR medium are: knockout (KO)-DMEM (Life Technologies) containing 20 % KO-serum replacement (KO-SR, Life Technologies), nonessential amino acids (NEAA), glutamine, penicillin/streptomycin, 0.1 mM 2- mercaptoethanol and 4 ng/ml fibroblast growth factor 2 (FGF2) (bFGF, R&D Systems Inc., Minneapolis, MN, USA). The medium was refreshed three times a week.

The hESC line H7 (46, XX) (WiCell Research Institute, Madison, WI, USA) was used as a comparison in the immunocytochemical stainings and PCR for pluripotency [7].

### Reprogramming of Fibroblasts to iPSCs

#### Plasmid Vectors

Vectors are available on Addgene repository upon request: 27,077 (pCXLE-hOCT3/4-shp53-F); 27,078 (pCXLE-hSK); 27,080 (pCXLE-hUL) and 37,624 (pCXWB-EBNA1) [8]. Plasmid DNA cocktail contained the four vectors in equimolar amounts (1:1:1:1).

#### Sendai Virus Reprogramming

150,000 fibroblasts were transduced with Sendai virus vectors following CytoTune®-iPS Sendai Reprogramming Kit’s guidelines, at a MOI of 1.25.

#### Lipofectamine Transfection

Fibroblasts at 80–90 % confluence growing in 6-well plates wells were transfected with 7.5 μl Lipofectamine 3000 reagent, 5 μl P3000 and 2.5 μg of plasmid DNA cocktail, following manufacturer’s instructions.

#### Neon Transfection System

Human fibroblasts (6 × 10^5^) were transfected with the Neon Transfection System (Life Technologies) according to manufacturer’s instructions. Conditions used were 1650 V, 10 ms, 3 time pulses, with a total of 3 μg plasmid DNA cocktail.

#### Nucleofector System

Human fibroblasts (6 × 10^5^) were transfected with the 4D-Nucleofector™ System (Lonza) according to manufacturer’s instruction, preset [EO114], with 3 μg plasmid DNA cocktail.

After Lipofectamine 3000, Neon or Nucleofector transfection and Sendai virus transduction, fibroblasts were cultured for 7 days in Fibroblast Medium, then trypsinized and re-plated onto 6-well plates wells layered with MEF feeder cells. The culture medium was switched the next day to KSR supplemented with bFGF.

### Characterization of iPSC Lines

#### DNA and RNA Extraction

Nucleospin Tissue XS kit (Macherey-Nagel) and RNeasy Mini Kit (Qiagen) have been respectively used to extract DNA and RNA, following manufacturers' instructions.

#### RT-PCR and PCR

Total RNA (1 μg) was reverse transcribed with High Capacity cDNA Reverse Transcription Kit (Life Technologies) and random hexamers, following the manufacturer’s protocol. RT reaction without Reverse Transcriptase enzyme served as internal control. PCR was performed on 30 ng cDNA or 25 ng gDNA, with primer pairs indicated in Table S1, with DyNAzyme II TAQ DNA Polymerase (Life Technologies). We used the H7 line as a positive control for pluripotency.

#### In Vitro Analysis of Pluripotency

The pluripotency of the iPSC lines UTA.11916.EURCSs, UTA.137062.EURCSp, UTA.107016.EURCAp and UTA.119024.EURCSp was verified by the formation of embryoid bodies (EBs). To form EBs, feeder cells were removed mechanically and iPSCs were scraped and placed into a suspension culture in EB medium (KO-DMEM with 20 % FBS, Non-Essential Amino Acid (NEAA), L-glutamine and penicillin/streptomycin). Medium was refreshed every 2 to 3 days, and EBs were cultured for 5 weeks after which RNA isolation and reverse transcription from the EBs was performed as described above. The expression of markers characteristic of ectoderm (PAX6 or SOX-1), endoderm (AFP or SOX-17), and mesoderm (KDR or ACTC1) development were studied from EBs and GAPDH was used an endogenous control. PCR primers for detecting the aforementioned genes are presented in Table S1.

#### Karyotyping

Genome-wide screening for gross chromosomal abnormalities was carried out with KaryoLite BoBs (Product number 4501–0010, Perkin Elmer) in the Finnish Microarray and Sequencing Centre, as described elsewhere [9]. The assay measures DNA copy numbers at the chromosome arm resolution, by evaluating the signal of bacterial artificial chromosomes (BACs) immobilized onto color-encoded polystyrene beads, detectable by a Luminex fluorometer. 97 individual beads cover p and q arms of all chromosomes 1–22, X and Y (q arms in acrocentric chromosomes) and can detect arm-specific aneuploidies in all 24 chromosomes in a single assay.

#### Immunocytochemistry

The cells were washed three times with room temperature PBS. After removing PBS, the cells were fixed for 20 min with room temperature 4 % PFA (Sigma Aldrich) to prevent cell detachment, and unspecific binding was blocked for 45 min with 1 % bovine serum albumin (BSA), 0.1 % Triton X-100 (Sigma Aldrich), 10 % normal donkey serum (NDS, Millipore) in 1X PBS. Blocking solution was removed with 1 % NDS, 0.1 % TritonX-100 and 1 % BSA in PBS 1X, then primary antibodies diluted in the same solution were incubated overnight at +4 °C.

The primary antibodies included pluripotency markers Nanog (Anti-human Nanog goat IgG; R&D Systems, Inc) 1:100; Oct-3/4 (Anti-human Oct.-3/4 goat IgG; R&D Systems, Inc.) 1:400; SOX-2 (SOX-2 goat IgG; Santa Cruz) 1:200; SSEA-4 (SSEA-4 mouse IgG; Santa Cruz) 1:100; TRA-1–60 (Anti-TRA 1–60 mouse IgM; Millipore) 1:200; and TRA 1–81 (Anti-TRA 1–81 mouse IgM; Millipore) 1:200. Secondary antibodies, 1:800 in 1 % BSA in PBS 1X, were incubated for 1 h at RT, protected from light, and included Alexa Fluor 568 nm donkey anti-goat IgG (Invitrogen) for Nanog, Oct-3/4 and Sox-2; Alexa Fluor 568 nm goat anti-mouse IgG H&L (Invitrogen) for SSEA-4; and Alexa Fluor 568 nm goat anti-mouse IgM M chain (Invitrogen) for TRA 1-60 and TRA 1-81. Samples were mounted with Vectashield Mounting Medium with DAPI (Vector Laboratories). Images were captured with fluorescence microscope (Olympus IX51) connected to a camera (Olympus DP30BW) and processed with DP manager software (Olympus).

## Results and Discussion

### Reprogramming Efficiency

The greatest hurdle in reprogramming terminally differentiated cells into iPSCs is reprogramming efficiency. Many methods of reprogramming are inefficient yielding iPSCs in much less than 1 % of the starting adult cells. To overcome this limitation many methods have been developed involving the use of viruses and/or chemicals to enhance reprogramming efficiency at the cost of safety and resources. The use of non-integrating viruses, such as the Sendai virus, delivering episomal vectors for reprogramming has enhanced the safety of the method maintaining a high reprogramming efficiency. Still, Sendai-based commercial kits are expensive and do require separate laboratory space (biosafety level 2) and dedicated equipment for virus work. Furthermore, the episomal vectors contained in the kits are patented and their sequence is not available, even though it is known what reprogramming factors they encode for. This also limits the research and testing of additional reprogramming cocktails. Non-integrating vectors are freely available and are amenable to being modified and/or used in custom formulations. They can be delivered by standard chemical transfection or electroporation, but these methods reportedly show lower efficiency rates.

Fibroblasts are the cell type most widely used in reprogramming. Even if other types of terminally differentiated cells have been shown to be more amenable for reprogramming than skin fibroblasts, the latter are easily collected and managed and remain nowadays the primary source of cells for reprogramming [9, 10].

We chose to test the most widely used methods for generating iPSC lines from dermal fibroblasts (hDF) of three different patients (hDF 107, 137 and 119), to compare their reprogramming efficiency and take into account the variability of real-world patients.

**Fig. 1:**
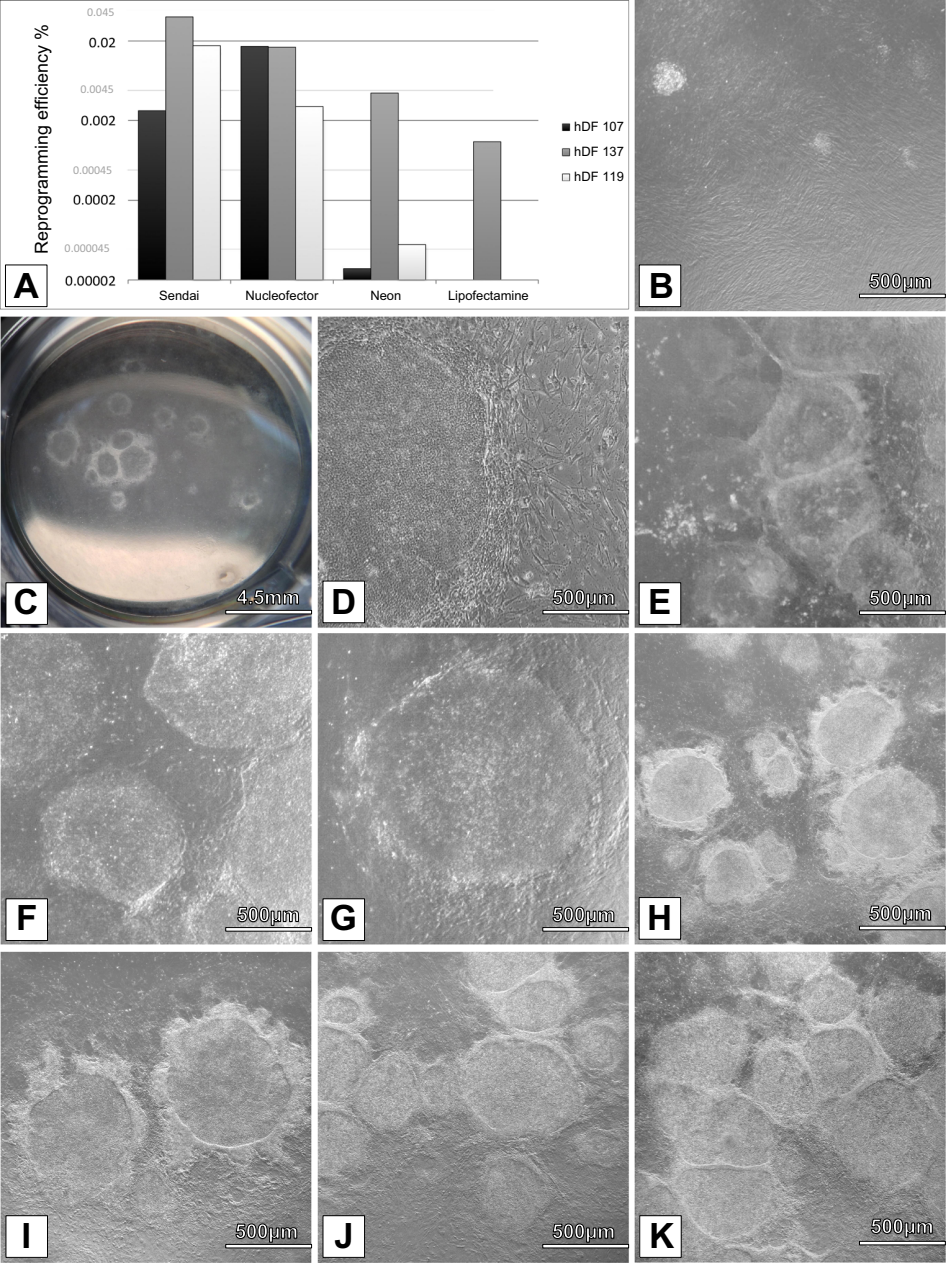
**Human fibroblast reprogramming.** Human dermal fibroblasts (hDF) from three different patients (hDF 107, 119 and 137) were reprogrammed into iPSC lines by using four different methods (Sendai virus, Nucleofector, Neon transfection system and Lipofectamine 3000) to deliver the reprogramming plasmids. Average reprogramming efficiency of the four methods used is presented for each hDF (**a**). Reprogramming efficiency is expressed in nascent colonies per cells transduced/transfected and the scale in the plot is logarithmic. Pictures of iPSCs colonies at different passage numbers are shown, each representative of a different condition: colonies are starting to grow on top of MEFs two weeks after transfection of hDF 137 (**b**), and colonies at passage 1 are shown in (**c**); Cytotune Sendai Reprogramming kit, at passage 9 from hDF 119 (**d**); Nucleofector Transfection System, at passage 4 from hDF 107 (**e**), hDF 119 (**f**) and hDF 137 (**g**); Neon Transfection System at passage 8 from hDF 107 (**h**), passage 5 from hDF 119 (**i**) and hDF 137 (**j**). iPSCs obtained from hDF 137, at passage 6 with Lipofectamine 3000 (**k**)

As expected, Sendai-virus based reprogramming was overall the most efficient method (0.0026 %, 0.04 % and 0.0173 % efficiency for hDFs 107, 137 and 119, respectively (Fig. 1A**)**. Nucleofector showed the most robust performance and good reprogramming efficiency (0.017 %, 0.0166 % and 0.003 %), compared to Neon electroporation (2.7·10^−5^%, 0.0044 % and 5.5·10^−5^% for hDFs 107, 137 and 119, respectively). As expected, the method based on chemical delivery (Lipofectamine 3000) showed the lowest reprogramming efficiency being able to successfully reprogram only hDF 137 (0.001 %). This is in line with the results obtained with the other methods as hDF 137 showed the highest overall reprogramming rate. It can be speculated that properly reprogrammed colonies could be obtained by raising the number of fibroblasts transfected. Colonies obtained with any of the methods showed similar phenotype (Fig. 1B-K).

Cell type and differentiation stage have been shown to influence reprogramming efficiency [11]. Epigenomic state, genetic variability and stochastic mechanisms all play a role in defining individual cell line’s response to reprogramming [12]. Notably, primary fibroblasts showed differences in the reprogramming efficiency, consistent between methods (Fig. 1). hDFs 107 and 119 showed decreased reprogramming efficiency, and failed to produce viable iPSCs-like colonies with Lipofectamine 3000 transfection in our experimental setting. hDF 137 was more amenable to reprogramming and produced colonies with good efficiency with all methods (0.04 %, 0.0166 %, 0.0044 and 0.001 % with Sendai, Nucleofector, Neon and Lipofectamine 3000 respectively). Interestingly, by using the Nucleofector we were able to efficiently reprogram hDF 107 at higher efficiency than by using any of the other methods..

**Fig. 2:**
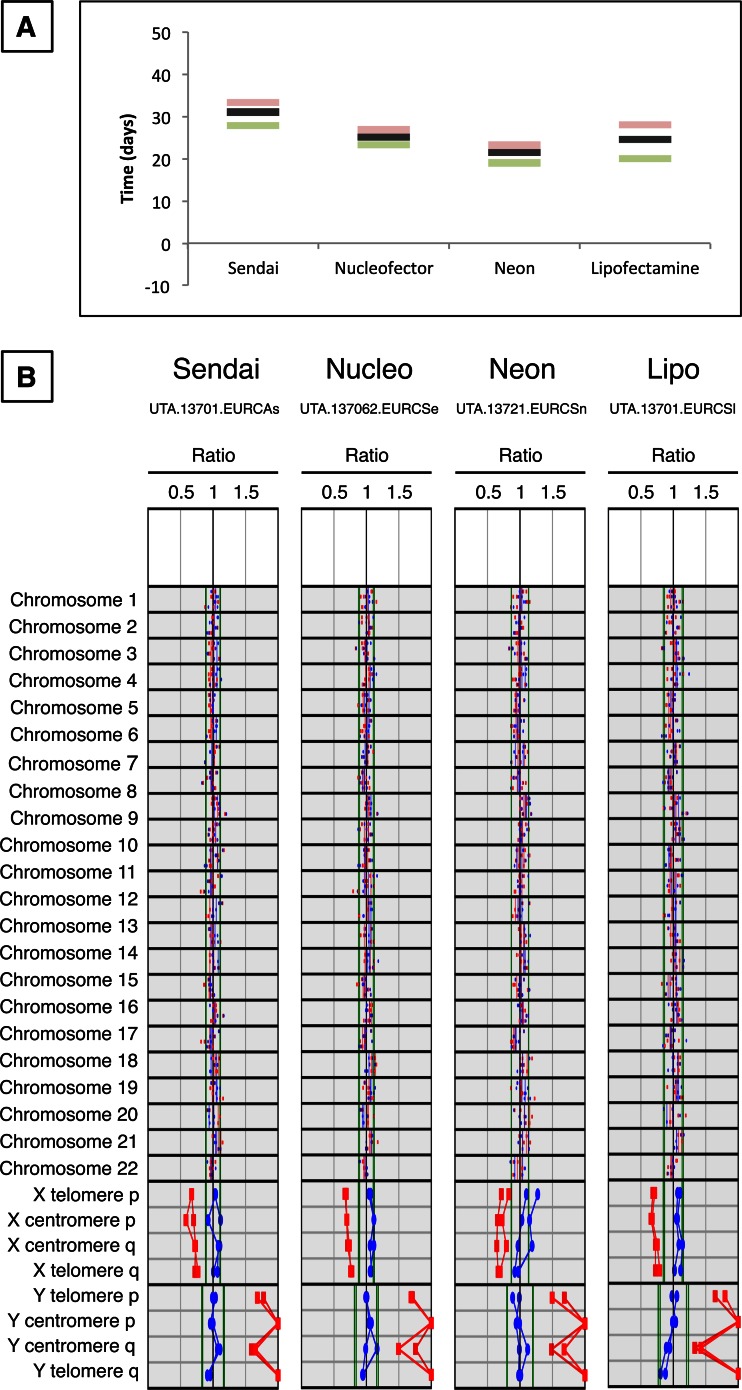
**iPSC colony picking times and karyotype analyses of four iPSC lines originating from one patient.** (**a**) Comparison of the average time (dark bar) from the initial transfection or transduction of the primary hDF cell lines to the picking day. Green bars show the earliest day of picking, red bars show the latest day for each method. The number of independent reprogramming rounds was three for each method, where at least 600,000 hDF per patient were lipofected, 900,000 hDF per patient were electroporated with either Neon or Nucleofector systems, and 150,000 hDF per patient were transduced with Sendai virus in each reprogramming. (**b**) Karyotype analyses of representative colonies, all derived from the same patient (hDF 137) with different methods, are shown. Red and blue dots indicate chromosomal signal ratios of sample DNA against female (red) and male (blue) reference normal karyotype DNA, as detected by KaryoLiteTM BoBs™ assay. Signal from normal chromosomes against both male and female references should lie inside the reference area around value 1, whereas with an abnormal karyotype both signals lie outside the reference area. A female probe pattern is defined when X and Y probe ratios are included in the expected range for a female sample (red line/dots inside and blue line/dots outside the normal expected X/Y range); a male pattern is defined by a reverse pattern (blue line/dots inside and red line/dots outside the normal expected X/Y range). Each plot shows the signal of two technical replicates of the same sample. All colonies show a normal male karyotype

Methods showed small differences in the average number of days when colonies appeared to be ready for picking (Fig. 2A). On average, most colonies were picked ~25 to ~30 days post transfection/transduction. Lipofectamine 3000 resulted in colonies which could be picked on average at day 24 post transfection, up to day 28; electroporation produced colonies which could be picked, on average, as early as 22–25 days post transfection, with Sendai-reprogrammed colonies trailing shortly after at day 29. The viable period for picking colonies, for each method, has been ~ ±3 days around the average picking day, with small differences between methods. We hypothesize that the variability of the individual operator could have a deeper impact than any other intrinsic variable.

### Gene Expression and Plasmid Contamination

Of all picked colonies, some failed to develop further upon replating onto feeder layer, or showed signs of differentiation or aberrant phenotype. The number of abortive colonies showed dependency on the method; Sendai virus resulted in 0 % to 20 % abortive colonies, Nucleofector transfection ranged from 30 % to 45 %, Neon transfection ranged from 0 % to 45 % and finally Lipofectamine from 33 % to 100 % (data not shown). Specifically, it was possible to obtain colony-like structures budding out of the feeder layer with Lipofectamine 3000 for hDFs 107 and 119, but they failed to develop further upon replating.

**Fig. 3:**
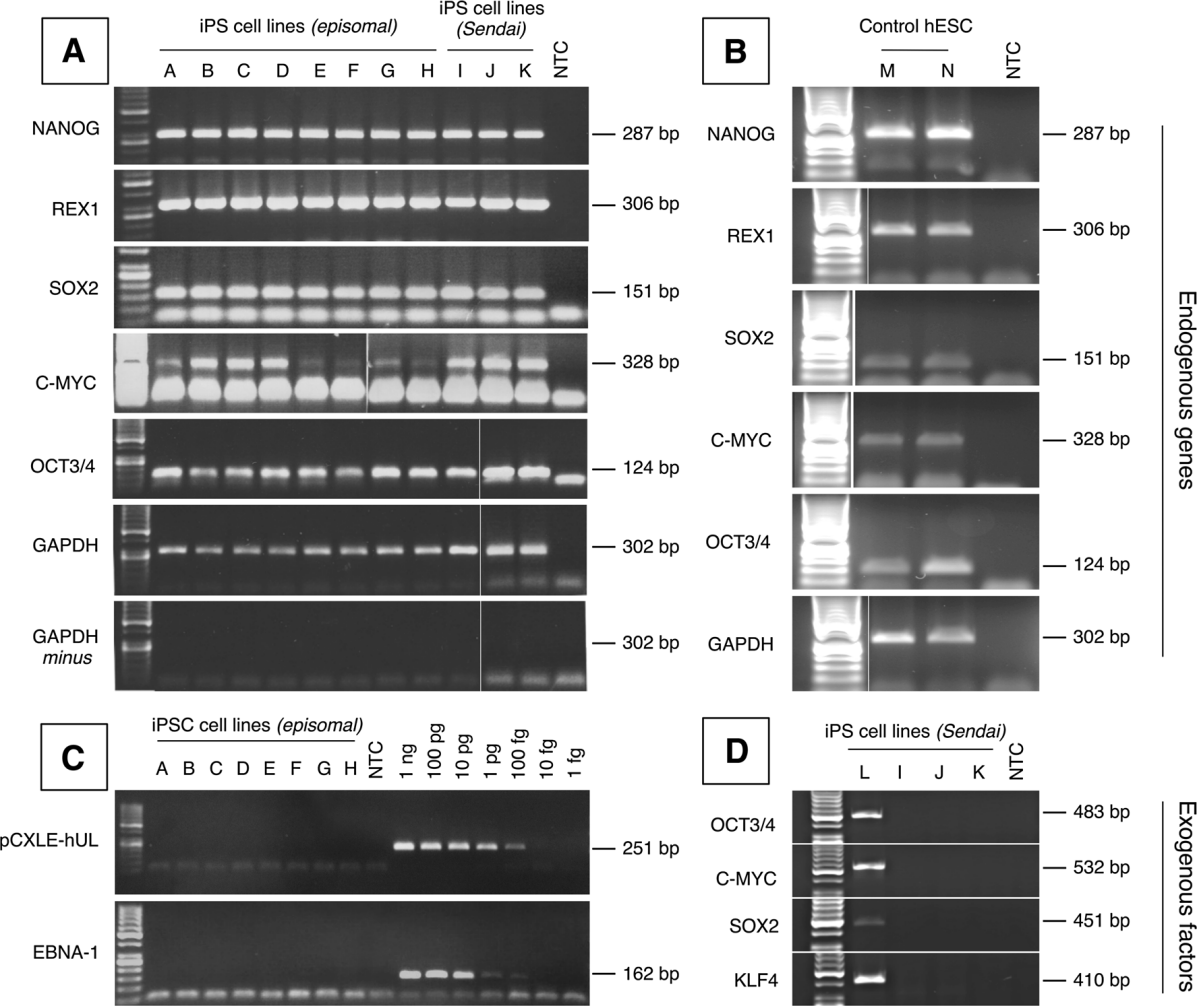
**Expression of pluripotency marker genes and absence of reprogramming plasmids in the iPSC lines.** Representative RT-PCR and PCR analyses for pluripotent cell markers and reprogramming vectors is shown. As a negative control, GAPDH amplification was also performed without reverse transcription. Total RNA and DNA was isolated from iPSC clones established with either Lipofectamine 3000 (lanes A-B), Neon (lanes C-E), Nucleofector Transfection System (lanes F-H) and CytoTune®-iPS Sendai Reprogramming Kit (lanes I-K). Results from hDF 107 (C, F, I), hDF 137 (A, B, D, G, J) and hDF 119 (E, H, K) derived iPSCs are shown. NANOG, REX, SOX, c-MYC and OCT3/4 gene fragments could be amplified from 30 ng cDNA, and GAPDH was used a loading control (**a**). Pluripotency genes expression was also assessed (**b**) in the commercially available hESC H7 line (lanes M-N). PCR failed to amplify plasmid DNA from both individual vectors (pCX-hUL) or EBNA-1 sequence from all vectors (**c**), as well as fragments from viral sequences (**d**) suggesting that all exogenous reprogramming agents are lost during iPSC passaging. DNA from early passages Sendai derived iPSCs was used as positive control (lane L)

Colonies that showed correct phenotype upon repeated passaging were selected for further characterization, each colony giving rise to an iPSC cell line. We investigated the expression of pluripotency genes in iPSCs cell lines with different passage numbers. All iPSCs lines tested expressed the pluripotency markers NANOG, REX1, SOX2, c-MYC and OCT3/4 (Fig. 3a). As an expression control, a commercially available human embryonic stem cell line H7 was used (Fig. 3b). A possible genomic DNA contamination in the RNA samples has been ruled out by primer design and by performing the PCR reaction on RNA without reverse transcription on the reference gene GAPDH (Fig. 3A) and on the pluripotency markers (data not shown).

**Fig. 4:**
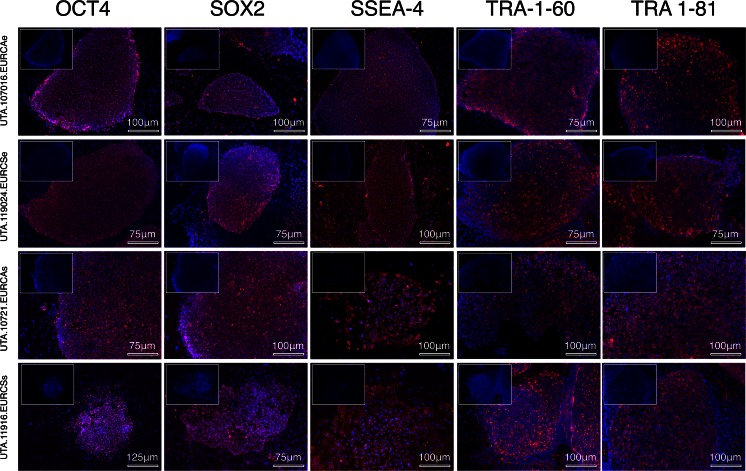
**Immunocytochemical staining for pluripotency markers of iPSCs colonies.** The expression of pluripotency genes in the iPSC lines was studied at the protein level by immunocytochemical staining. Images are composite of DAPI (blue) and specific signal (red) for OCT-4, SOX2, SSEA-4, TRA 1-60 and TRA 1-81 pluripotency markers. DAPI signal alone is shown, as a reference for each staining, in the upper left corner

Furthermore, we confirmed by immunocytochemical staining the expression of completely reprogrammed human pluripotent stem cell markers. iPSC colonies were found positive for OCT4, SOX2, SSEA-4, TRA 1–60 and TRA 1–81 antigens, without noticeable differences between methods (Fig. 4) or when compared to H7 hESC line (Fig. S1).

Next, we wanted to estimate plasmid loss. All episomal plasmids encode for EBNA-1, an Epstein-Barr virus derived protein, which can enhance the maintenance of circular DNA vectors harboring an OriP sequence in eukaryotic nuclei [13]. It is estimated that such episomal vectors have 2 % to 5 % chance of being lost at each cell division [14]. Some authors report that plasmids are not detectable at passages 11 to 20 (~80–120 days after transfection) in the vast majority of the colonies tested [8]. We failed to detect EBNA1 DNA in all iPSCs cell lines at passages 7 to 9 (~74–88 days after transfection) (Fig. 3C), indicating that all exogenous reprogramming agents are lost during iPSC passaging. iPSCs generated by the Sendai virus method were investigated by PCR at passage 15, and failed to amplify viral genomes as well (Fig. 3D).

The pluripotency of the iPSC lines UTA.11916.EURCSs, UTA.137062.EURCSp, UTA.107016.EURCAp and UTA.119024.EURCSp was verified by EB formation and the EBs were shown to express at least one marker from each of the three germ layers (endoderm, ectoderm and mesoderm) (Fig. S2).

### Genomic Integrity

Karyotypic abnormalities occur without notable differences in hESCs and iPSCs, and are commonly observed as early as three passages after derivation [15]. The maintenance of a correct karyotype is crucial for the exploitation of pluripotent stem cell lines in the clinical setting, to avoid flaws in the reproducibility of the results but more importantly, to prevent dysregulation of physiological pathways and cellular processes related to tumorigenicity. The most common karyotypic abnormalities in hESCs involve copy number variations of whole chromosomes or of telomeric regions of chromosomes [15, 16].

To detect chromosomal abnormalities in our lines, we used a BACs-on-beads (BoBs) method employed for the genetic evaluation of the products of conception, which also has proven useful for the genetic characterization of stem cell lines [17, 18]. The assay can detect arm-specific aneuploidies in all 24 chromosomes in a single assay and it is expected to cover most gross abnormalities. We performed two technical replicates of three different iPSC lines per method, and did not find any chromosomal rearrangements (Fig. 2B). It has been reported that most abnormalities occur from passage 3 to ~120, with a ~ 10 % chance of finding an aneuploidy line, with small fluctuations depending on the method being used, possibly hinting at a mutagenic role of the method itself [15, 19]. These reports gather data from hundreds or thousands of cell lines and the lack of chromosomal rearrangements in our experimental setting could be due to the small number of lines analyzed or the relatively low passage number. Our results suggest that very low rates of karyotypic abnormalities are to be expected in routine experiments when performing established reprogramming protocols.

Efficiency of each method aside, we did not observe method-specific differences or patterns at gene or protein marker expression levels; all lines tested were also karyotypically normal. This observation is in line with other reports, which compared other methods for iPSCs production [19, 20]. We produced and characterized a number of patient-specific iPSC lines, which exceeds what is usually required for experiments in a standard laboratory setting. With increasing number of iPSC lines, or when culturing them for a longer period of time, it can be expected that differences in the marker expression or aneuploidies arise [15]. However, the vast majority of the iPSC lines we produced expressed a wide range of pluripotency markers at both gene and protein levels, with a normal karyotype. We used non-commercial hDFs from three different patients, and these hDFs showed differences in the reprogramming efficiency, consistent between methods. However, analyses from iPSCs derived with different methods from the same patient were fairly comparable.

Lipofectamine 3000 can be used in a wider number of laboratories, as it does not require dedicated equipment but reprogramming efficiency was highly unreliable and extremely dependent on the hDF intrinsic amenability to reprogramming. Viral reprogramming is time-efficient but still poses questions to the use of generated iPSCs in the clinical setting, requires dedicated labware and hoods and forbids the use of custom cocktails by using proprietary reagents. Electroporation, although more time consuming and needing dedicated equipment, allows an efficient production of iPSCs by rivalling with viral efficiency.

Our results directly apply to hDF reprogramming and we concentrate on comparing different reprogramming methods as well as pointing out the individuality of each patients' cells being reprogrammed. iPSCs can be produced from a large variety of other somatic cells than hDFs like peripheral blood mononuclear cells, keratinocytes, adipose cells or mesenchymal stem cells, and further studies would be needed to compare the efficiency of current methods to reprogram those other cell types.

## Conclusions

In this study we compared four methods, which use three different approaches (virus-, electroporation- and chemical-based) to deliver reprogramming factors to hDFs from three patients and reprogram them to iPSCs. We showed that Sendai virus method, as expected, achieved the overall highest reprogramming rate. Electroporation-based methods followed with Nucleofector performing better than Neon system in our experimental conditions. Lipofectamine 3000 delivery resulted in the lowest reprogramming rate and could only generate iPSCs out of one of the three patient-derived hDFs. Differences in reprogramming rates of fibroblasts taken from different patients showed consistency between methods. Finally, characterized iPSCs did not reveal any significant differences in their morphology, expression of pluripotency markers, karyotype or gene expression profiles, regardless of the method employed. Furthermore, they were free of the episomal vectors used in the reprogramming.

Taken together, these results suggest that iPSCs colonies obtained with any of the methods tested here display the expected iPSCs-like phenotype, express a broad range of pluripotency markers at both mRNA and protein level and are free of the episomal reprogramming vectors. Moreover, karyotypic abnormalities are expected to occur with low frequency regardless of the reprogramming method employed.

## Electronic supplementary material

Supplemental information includes two figures and one table and can be found with this article online.

Figure S1 (DOCX 1.63 mb)

Figure S2 (DOCX 765 kb)

Table S1 (DOCX 18.0 kb)
