# Supplementary material for: A Comparative View on Easy to Deploy non-Integrating Methods for Patient-Specific iPSC Production
Source: Stem Cell Rev. 2015 Sep 5;11(6):900–8. doi: 10.1007/s12015-015-9619-3 (PMC4653244; doi:10.1007/s12015-015-9619-3)
Supplement: Supplementary file 1 — (DOCX 1.63 mb) [file 12015_2015_9619_MOESM1_ESM.docx]

**Supplemental Figures and legends**

**
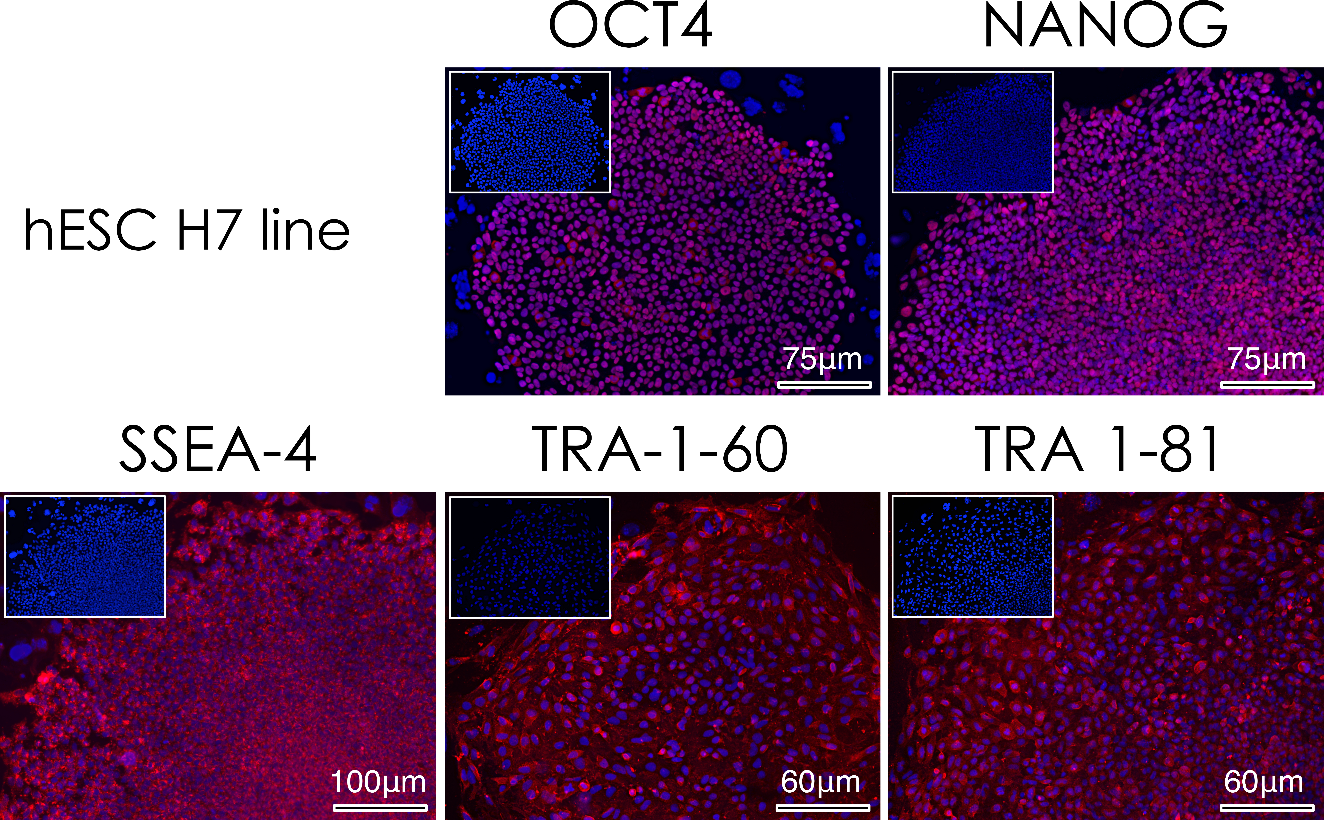
**

**Figure S1.** **Immunocytochemical staining for pluripotency markers of human embryonic stem cell (hESC) line H7**. **Related to Figure 4.** The expression of pluripotency genes in the commercially available hESC line H7 was studied at the protein level by immunocytochemical staining. Images are composite of DAPI (blue) and specific signal (red) for OCT-4, SOX2, SSEA-4 TRA 1-60 and TRA 1-81 pluripotency markers. DAPI signal alone is shown, as a reference for each staining, in the upper left corner.
