# Supplementary material for: A Comparative View on Easy to Deploy non-Integrating Methods for Patient-Specific iPSC Production
Source: Stem Cell Rev. 2015 Sep 5;11(6):900–8. doi: 10.1007/s12015-015-9619-3 (PMC4653244; doi:10.1007/s12015-015-9619-3)
Supplement: Supplementary file 2 — (DOCX 765 kb) [file 12015_2015_9619_MOESM2_ESM.docx]

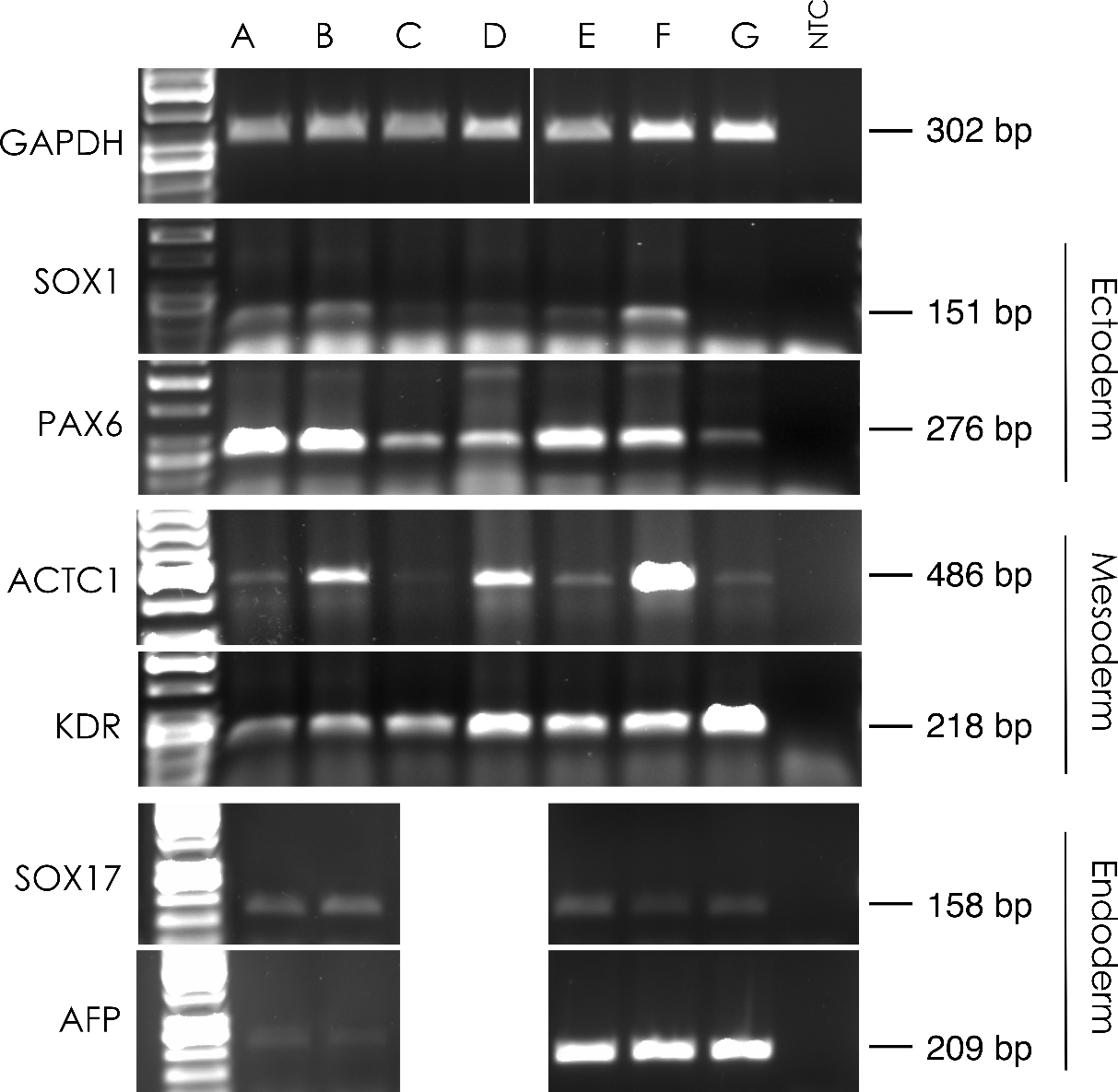


**Figure S2**. **The expression of marker genes of each germ layer (endoderm, ectoderm and mesoderm) in the embryoid body (EB)-assay.** Representative PCR analyses for germ layer specific gene expression markers is shown. Total RNA was isolated from EBs produced from iPSCs reprogrammed with either CytoTune®-iPS Sendai Reprogramming Kit (lanes A-B) or Nucleofector Transfection System (lanes C-G; hDF 107 lanes C,E; hDF 137 lane F; hDF 119 lane D,G). Germ layer specific PAX6, SOX-1 (ectoderm), AFP, SOX-17 (endoderm), KDR and ACTC1 (mesoderm) gene fragments were detected, and GAPDH was used as an endogenous control gene.
