# Supplementary material for: A Comparative View on Easy to Deploy non-Integrating Methods for Patient-Specific iPSC Production
Source: Stem Cell Rev. 2015 Sep 5;11(6):900–8. doi: 10.1007/s12015-015-9619-3 (PMC4653244; doi:10.1007/s12015-015-9619-3)
Supplement: Supplementary file 3 — (DOCX 18.0 kb) [file 12015_2015_9619_MOESM3_ESM.docx]

# **Table S1**. Primer sequences for detecting endogenous pluripotency gene expression, exogenous plasmids/Sendai vectors as well as for detecting the three different germ layers produced in EB-assay.

| **Gene** | **Forward Primer 5’-3’** | | **Reverse Primer 5’-3’** | **Amplicon Size** |
| --- | --- | --- | --- | --- |
| **Pluripotency markers** | | |  |  |
| OCT3/4 | | gacagggggaggggaggagctagg | cttccctccaaccagttgccccaaac | 144 |
| c-MYC | | gcgtcctgggaagggagatccggagc | ttgaggggcatcgtcgcgggaggctg | 328 |
| NANOG | | TGCAAATGTCTTCTGCTGAGAT | GTTCAGGATGTTGGAGAGTTC | 287 |
| SOX2 | | gggaaatgggaggggtgcaaaagagg | ttgcgtgagtgtggatgggattggtg | 151 |
| REX1 | | cagatcctaaacagctcgcagaat | gcgtacgcaaattaaagtccaga | 306 |
| **Plasmid detection** | | |  |  |
| EBNA-1 | | GGGGTAGAGGACGTGAAAGA | GGTGGAAAAATGGCCTTCTA | 162 |
| OCT3/4  *(pCXLE-hOCT3/4-shp53-F)* | | CATTCAAACTGAGGTAAGGG | TAGCGTAAAAGGAGCAACATAG | 124 |
| LIN28  *(pCXLE-hUL)* | | AGCCATATGGTAGCCTCATGTCCGC | TAGCGTAAAAGGAGCAACATAG | 251 |
| SOX2  *(pCXLE-hSK)* | | TTCACATGTCCCAGCACTACCAGA | TTTGTTTGACAGGAGCGACAAT | 111 |
| **Sendai vectors detection** | | |  |  |
| SOX2 | | ATGCACCGCTACGCAGTGAGCGC | AATGTATCGAAGGTGCTCAA | 451 |
| OCT3/4 | | CCCGAAAGAGAAAGCGAACCA | AATGTATCGAAGGTGCTCAA | 483 |
| KLF4 | | TTCCTGCATGCCAGAGGAGCCC | AATGTATCGAAGGTGCTCAA | 410 |
| c-MYC | | TAACTGACTAGCAGGCTTGTCG | TCCACATACAGTCCTGGATGATGATG | 532 |
| **EB primers**  **Endodermal** | | |  |  |
| AFP | | CATCCAGGAGAGCCAAGCAT | CGCCACAGGCCAATAGTTTG | 209 |
| SOX17 | | CGCACGGAATTTGAACAGTA | CACACGTCAGGATAGTTGCAG | 166 |
| **Ectodermal** | | |  |  |
| PAX6 | | AACAGACACAGCCCTCACAAACA | CGGGAACTTGAACTGGAACTGAC | 275 |
| SOX1 | | AAAGTCAAAACGAGGCGAGA | AAGTGCTTGGACCTGCCTTA | 158 |
| **Mesodermal** | | |  |  |
| KDR | | GTGACCAACATGGAGTCGTG | TGCTTCACAGAAGACCATGC | 218 |
| ACTC1 | | GGAGTTATGGTGGGTATGGGTC | AGTGGTGACAAAGGAGTAGCCA | 486 |
| **Housekeeping** | | |  |  |
| GAPDH | | AGCCACATCGCTCAGACACC | GTACTCAGCGCCAGCATCG | 302 |
|  | |  |  |  |

**EB =** Embyonic Body**; OCT3/4** = POU Class 5 Homeobox 1; **c-MYC** = V-Myc Avian Myelocytomatosis Viral Oncogene Homolog1; **NANOG** = Homeobox Transcription Factor Nanog; **SOX2** = SRY (sex determining region Y)-box 2; **REX1** = RNA Exonuclease 1 Homolog (S. Cerevisiae); **EBNA-1** = Epstein–Barr nuclear antigen 1; **LIN28** = RNA-Binding Protein LIN-28; **KLF4** = Kruppel-Like Factor 4; AFP = alpha-fetoprotein; **SOX-17** = SRY (sex determining region Y)-box 17; **PAX6** = paired box 6; **SOX1** = SRY (sex determining region Y)-box 1; **KDR** = kinase insert domain receptor; **ACTC1** = actin, alpha, cardiac muscle 1; **GAPDH** = glyceraldehyde-3-phosphate dehydrogenase
